# Seed coat colour of Indian mustard [*Brassica juncea* (L.) Czern. and Coss.] is associated with *Bju.TT8* homologs identifiable by targeted functional markers

**DOI:** 10.3389/fpls.2022.1012368

**Published:** 2022-10-05

**Authors:** Manoj Kumar Patel, Rajat Chaudhary, Yashpal Taak, Priya Pardeshi, Joghee Nanjundan, K. K. Vinod, Navinder Saini, Sujata Vasudev, D. K. Yadava

**Affiliations:** ^1^ Division of Genetics, ICAR-Indian Agricultural Research Institute, New Delhi, India; ^2^ Indian Council of Agricultural Research (ICAR)- Indian Agricultural Research Institute, Regional Research Station, Wellington, India

**Keywords:** *Bju.ATT8*, *Bju.BTT8*, duplicate gene action, functional markers, Indian mustard, maternal-effect, seed coat colour

## Abstract

Seed coat colour is an important trait in Indian mustard. Breeding for seed coat colour needs precise knowledge of mode of inheritance and markers linked to it. The present study was focussed on genetics and development of functional markers for seed coat colour. F_1_s (direct and reciprocal) and F_2_ populations were developed by crossing two contrasting parents for seed coat colour (DRMRIJ-31, brown seeded and RLC-3, yellow seeded). Phenotypic results have shown that the seed coat colour trait was under the influence of maternal effect and controlled by digenic-duplicate gene action. Further, *Bju.TT8* homologs of both parents (DRMRIJ-31 and RLC-3) were cloned and sequenced. Sequencing results of *Bju.TT8* homologs revealed that in RLC-3, gene *Bju.ATT8* had an insertion of 1279bp in the 7^th^ exon; whereas, gene *Bju.BTT8* had an SNP (C→T) in the 7^th^ exon. These two mutations were found to be associated with yellow seed coat colour. Using sequence information, functional markers were developed for both *Bju.TT8* homologs, validated on F_2_ population and were found highly reliable with no recombination between the markers and the phenotype. Further, these markers were subjected to a germplasm assembly of Indian mustard, and their allelic combination for the seed coat colour genes has been elucidated. The comparative genomics of *TT8* genes revealed high degree of similarity between and across the *Brassica* species, and the respective diploid progenitors in tetraploid *Brassica* species are the possible donors of *TT8* homologs. This study will help in the marker-assisted breeding for seed coat colour, and aid in understanding seed coat colour genetics more precisely.

## Introduction

Indian mustard [*Brassica juncea* (L.) Czern. and Coss., AABB; 2n=36] is one of the major oilseed crops and is globally cultivated in tropical and subtropical regions including the Indian sub-continent. In India, it is a predominant species among the rapeseed-mustard group and accounts for more than 90% of its total acreage (Chand et al., 2021; Yadava et al., 2022). Based on seed coat colour, Indian mustard is broadly categorized into two classes *viz.*, brown and yellow seeded. However, most Indian cultivars come under the brown seeded types (Padmaja et al., 2005). Seed coat colour in *B. juncea* is an important trait related to oil and seed meal quality. Yellow seeded Brassicas are known to contain more oil, protein and low fibre content than the brown seeded ones (Woods, 1980; Rahman and McVetty, 2011). Seed coat colour also affects the earliness in seed germination (Zhang et al., 2015). There are reports about poor storability of yellow seeded mustard than brown seeded due to thin testa leading to imbibitional damage (Zhang et al., 2006; Swami et al., 2016; Yadav et al., 2020). Therefore, there is an urgent need to improve the storability of Indian mustard which could be met by developing brown seeded cultivars.

The seed coat is developed from the differentiation of the integuments and chalaza tissues of the ovule after fertilization. In crucifers, accumulation of proanthocyanidin (oxidized form of flavonoids) in inner integuments of seed coat gives rise to brown pigmentation (Lepiniec et al., 2006) and mutations in the pathway genes lead to failure of proanthocyanidin production. In *B. juncea*, the brown seed coat colour is dominant over the yellow type, however, environmental conditions, maternal effect, and epistasis make its expression complex and varying (Mahmood et al., 2005). Further, several previous studies on the inheritance pattern and the number of genes governing the seed coat colour in *B. juncea*, indicate ambiguous results, suggesting a complex inheritance pattern. However, reports on monogenic (2016; Xu et al., 2010; Huang et al., 2012), digenic (Padmaja et al., 2005; Yan et al., 2009) and multigenic (Mahmood et al., 2005) inheritance are available in the literature. By mapping, QTLs controlling seed coat colour in *B. rapa* are reported later (Rahman et al., 2014; Zhang et al., 2019). Earlier, seed coat phenotypes of a series of *Arabidopsis thaliana* mutants, *transparent testa* (*TT*), having varying seed colour has been reported by Debeaujon et al. (2000). Among these mutants, Nesi et al. (2000) identified that the mutant gene of *TT8* produces an aberrant protein affecting the expression of two other genes, *dihydroflavonol 4-reductase* (*DFR*) and *Banyuls* (*BAN*), involved in flavonoid metabolism in interaction with genes such as *TT2*, and *TTG1*. Subsequently, Baudry et al. (2004) identified that these three synergistically acting genes regulate proanthocyanidin biosynthesis in *Arabidopsis*. The role of *TT8* in determining seed coat colour in *B. rapa* was later reported (Li et al., 2012; Niu et al., 2020), and also in *B. juncea* (Padmaja et al., 2014). They reported two homologs of the *TT8* gene are to be responsible for seed coat colour development. Recently, Zhai et al. (2020) reported the role of *BnTT8* homologs in seed coat colour development in *B. napus*, through targeted mutagenesis of *BnTT8* homologs that leads to production of yellow seed coat colour coupled with increased seed oil and protein content. In addition, Shen et al. (2021) have also suggested the role of *TT8* gene governing seed coat colour in different *Brassica species* through metabolite profiling and transcriptome analysis. However, *TT2* gene is also reported to be involved in flavonoid biosynthesis that leads to seed coat colour formation in *B. napus* (Zhou et al., 2016; Xie et al., 2020).

DNA-based markers are indispensable for marker-assisted breeding as they facilitate the selection of the trait, irrespective of plant growth stages, environments, and even in the absence of phenotypic expression. In *B. juncea*, Negi et al. (2000) found linked AFLP markers for seed coat colour and were converted into SCAR markers using the bulked segregant analysis (BSA) approach in the F_4_ generation. Subsequently, using RFLP markers, three QTLs for coat colour variation *viz. SC-B04*, *SC-A10*, and *SC-A06* were reported in a doubled haploid (DH) population derived from brown-seeded cultivar ‘RLM-514’ and a yellow-seeded breeding line (Mahmood et al., 2005). At the same time, Padmaja et al. (2005), identified two genes, *BjSC1* and *BjSC2* linked with SSR markers Na10-A08 and Ni4-F11, respectively. Although, Padmaja et al. (2014) has reported two independent markers targeting both the homologs of *TT8* genes in *B. juncea*, but required more operational time that are poorly suitable in marker assisted selection. Recently, Huang et al. (2016) used intron polymorphism (IP) and sequence characterised amplified region (SCAR) markers to map linked genomic regions to seed coat colour using backcross inbred lines, and found that marker IP4 and Y1 were located on either side of yellow seed colour gene. In addition, seed coat colour gene, *Brsc1*fine-mapped employing whole genome re-sequencing coupled with BSA was reported by Wang et al. (2016). Besides, two co-localized major QTLs, *qSC9.1* and *qSCb9.1*, and one minor QTL, *qSC3.1* were also reported for seed coat colour variation in *B. rapa* (Zhang et al., 2019). Despite these limited reports, functional markers for seed coat colour that work across the genetic backgrounds and are useable in marker-assisted crop improvement in *B. juncea* have not yet been developed. To address this, we have developed functional markers targeting the *Bju.TT8* homologs and attempted to validate the same in a segregating and a germplasm assembly. The same set of germplasm assembly was evaluated using developed functional markers to deduce allelic pattern for *Bju.TT8* genes.

## Materials and methods

### Plant materials and phenotypic evaluation

For studying the variability for seed coat colour, a set of 127 diverse genotypes were screened and two contrasting varieties *viz.*, DRMRIJ-31 and RLC-3 were selected and crossed. DRMRIJ-31 was a conventional, high yielding, brown seeded cultivar developed from the directorate of Rapeseed and Mustard, Bharatpur, India and RLC-3 was a double zero canola quality, having yellow seeded and originated from Punjab Agricultural University, Ludhiana, Punjab. The F_1_s (both direct and reciprocal) were developed during *rabi* 2018-19. Grown during *rabi* 2019-20, the F_1_s were selfed to develop F_2_ seeds. Two rows of each parent and 15 rows of the F_2_ population were raised *rabi* 2020-21. Each row was 5m in length, with a row-to-row spacing of 45 cm and plant to plant distance of 15 cm. All the recommended agricultural operations such as thinning, weeding, irrigation, and fertilizer application were done as per recommended package of practices for better crop establishment. The seed coat colour of 526 F_2_ individual plants (F_3_ seeds) was scored visually and broadly categorized into two classes *i.e.*, brown and yellow.

### Mode of inheritance and marker-phenotype association

The maternal-effect was examined by observing the seed coat colour differences between direct and reciprocal F_1_ crosses. Inheritance pattern was determined by studying the segregation of F_3_ seeds for coat colour. The gene action and marker-phenotype association were analysed using the *χ^2^*-test (Pearson, 1916). The *χ^2^*-calculated value was compared with *χ^2^*-tabulated value at a 5% level of significance with (n-1) degrees of freedom. If *χ^2^*-calculated value is less than the *χ^2^*-tabulated value then it concludes that markers and phenotype are following expected segregation and *vice-versa*. The *χ^2^*-test can be done using the following formula:


$$x^{2}=\sum_{i=1}^{n}\frac{{\left(O_{i}-E_{i}\right)}^{2}}{E_{i}}\;$$

where, *O_i_* and *E_i_* represent the observed and expected frequency of each class, respectively.

### Molecular analysis

Approximately 300 mg of fresh tender leaves from each 127 genotypes, including individual F_2_ plants were used for DNA isolation using the CTAB method (Doyle and Doyle, 1987). DNA was quantified employing nanodrop (NanoDrop 2000, Thermo Scientific, USA) and quality was evaluated using 0.8% of agarose on gel electrophoresis. The DNA was stored at -20°C. For one PCR sample, a reaction mixture of volume 15µl was used in a thermal cycler (MiniAmp Plus, Applied biosystem) using ~50ng DNA template, 1.5µl 10x buffer S, 750 µM dNTP, 1U *Taq*-polymerase (Vivantis) and DNA primers 10 pM each (forward and reverse) were used. The PCR profile contained 40 cycles with customised profiles for each marker. The amplicons were resolved in 2.5% Agarose gel in a gel electrophoresis system; visualised and pictographed in a gel documentation system (SYNGENE), under ultra-violet transillumination.

### Sequencing of *Bju.TT8* homologs and development of markers

Full-length *Bju.TT8* genes were amplified in DRMRIJ-31 (brown seeded parent) and RLC-3 (yellow seeded parent) using primers TT8-RsF1 (5’-ggaaagtgatcggggctgagaaag-3’) and TT8-RsR4 (5’-ctcaaaatgattgtcgttcaaggca-3’) for *Bju.ATT8* gene, TT8-NsF1 (5’-aggtatggaaagtgatcggagctgagg-3’) and TT8-NsMPR (5’-catgttcttctctaaagttggcatcag-3’) for *Bju.BTT8* gene (Padmaja et al., 2014). Amplified products were cloned in pGEM^®^-T Easy vector (www.promega.com) and sequenced using Sanger method (Applied Biosystems). Using this sequencing information, the PCR-based markers were developed for both the homologs of *Bju.TT8* gene using online tool PrimerQuest™ (https://www.idtdna.com/PrimerQuest).

### Validation of *Bju.TT8* allelic combinations

526 F_2_ plants were used to validate functional markers for seed coat colour by studying segregation pattern with the phenotype. In addition, a germplasm assembly of 130 genotypes, which includes a diverse panel of 127 genotypes of *B. juncea*, two genotypes of *B. rapa* (2n=20; AA) *i.e.*, TL-15 (brown seeded), Pusa gold (yellow seeded), and one brown seeded *B. nigra* (2n=16; BB) genotype (IC-281862), were screened for seed coat colour alleles. Further, these genotypes were categorised into four different allelic combinations of *Bju.TT8* genes *i.e.*, AABB, AAbb, aaBB, and aabb among *B. juncea* genotypes.

### Comparative genomics of *TT8 *sequences

Available coding (CDS) and protein sequences of *TT8* homologs and orthologs in different *Brassica* species were downloaded from NCBI Genbank (https://www.ncbi.nlm.nih.gov). *TT8* gene sequences of *B. juncea* cv. Varuna and Heera were taken from Padmaja et al. (2014). In some *Brassica* species, where the *TT8* gene has not been reported, predicted CDS and protein sequences were obtained through NCBI blast (https://blast.ncbi.nlm.nih.gov/Blast.cgi) and an online software *Fgenesh+* (Solovyev, 2007), using the *TT8* protein sequence of *Arabidopsis thaliana* (NM_117050.3, Mayer et al., 1999) as reference. The CDS and protein sequences of *TT8* genes of different Brassicas *i*.*e*., *B. rapa* (XM_009115326.3; GU255866.1 and HQ337791.1, Wang et al., 2012)*, B. nigra* (cv. YZ12151 and Sangam)*, B. oleracea* (GU255867.1 and GU219990.1, Chiu et al., 2010)*, B. napus* (NM_001315974.2, Zhai et al., 2020; GU255864.1; GU255865.1; cv. Da-Ae)*, B. juncea* (KJ942581.1, Xie et al., 2014; cv. Varuna; DRMRIJ-31; RLC-3; Heera; AU-213; Sichuan Huangzi and T84-66)*, B. carinata* (cv. sxm20200214) and *A. thaliana* (NM_117050.3, Mayer et al., 1999) were aligned using the online tool, *Clustal Omega* (Sievers et al., 2011).

## Results

### Inheritance pattern of seed coat colour using phenotypic evaluation

**Figure 1:**
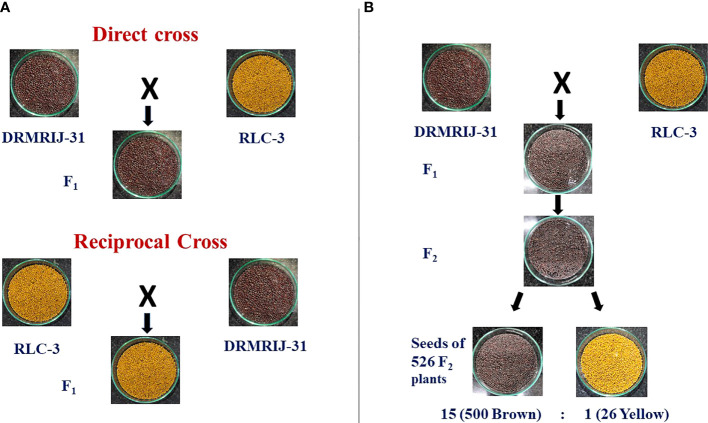
Inheritance pattern of seed coat colour **(A)** Seed coat colour differences in direct and reciprocal crosses of DRMRIJ-31 and RLC-3 **(B)** Duplicate dominant type segregation of seed coat colour in seeds of F_2_ plants.

Parents DRMRIJ-31 and RLC-3 were used to produce reciprocal F_1_ crosses. The F_1_ seeds harvested from DRMRIJ-31 × RLC3 cross were brown in colour, whereas the yellow seeds were obtained from RLC-3 × DRMRIJ-31 cross (**Figure 1A**). Since the seed coat colour exhibited maternal effect, the F_3_ seeds harvested from 526 F_2_ plants segregated into 500 plants producing brown seeds, and 26 plants producing yellow seeds (**Figure 1B**, **Supplementary Figure 1**, **Table 1A**). The observed values well agreed with 15:1 ratio for duplicate dominant gene action, involving two genes. The *χ^2^*-test showed that was a non-significant difference at 5% level of significance with a probability of 0.22 (**Table 1A**).

**Table 1:** Segregation analysis of seed coat colour and functional markers in F_2_-population using *χ^2^*-test.

S.N.	Particulars	Coat colour Classes	Ratio	Observed value	Expected Value	χ^2^-cal	χ^2^-tab(0.05, n-1 df)	P(0.05)
A.	Seed coat colour classes	Brown	15	500	493.125			
		Yellow	1	26	32.875			
		Total	16	526	526	1.53	3.84	0.22
B.	*Bju.ATT8* Marker Classes (Gene A)	*AA*	1	129	131.5			
		*Aa*	2	269	263			
		*aa*	1	128	131.5			
		Total	4	526	526	0.28	5.99	0.87
C.	*Bju.BTT8* Marker Classes (Gene B)	*BB*	1	133	131.5			
		*Bb*	2	247	263			
		*bb*	1	146	131.5			
		Total	4	526	526	2.59	5.99	0.27
D.	Combined marker segregation	*AABB* (Brown)	1	34	32.875			
		*AABb* (Brown)	2	61	65.75			
		*AAbb* (Brown)	1	34	32.875			
		*AaBB* (Brown)	2	61	65.75			
		*AaBb* (Brown)	4	122	131.5			
		*Aabb* (Brown)	2	86	65.75			
		*aaBB* (Brown)	1	38	32.875			
		*aaBb* (Brown)	2	64	65.75			
		*aabb* (Yellow)	1	26	32.875			
		Total	16	526	526	9.97	15.51	0.27
E.	Joint Classes of trait phenotype and *Bju.ATT8* marker	AA, Brown	4	129	131.5			
		Aa, Brown	8	269	263			
		aa, Brown	3	102	98.625			
		aa, Yellow	1	26	32.875			
		Total	16	526	526	1.73	7.81	0.63
F.	Joint Classes of trait phenotype and *Bju.BTT8* marker	BB, Brown	4	133	131.5			
		Bb, Brown	8	247	263			
		bb, Brown	3	120	98.625			
		bb, Yellow	1	26	32.875			
		Total	16	526	526	7.06	7.81	0.07

### Sequencing of *Bju.TT8* genes

**Figure 2:**
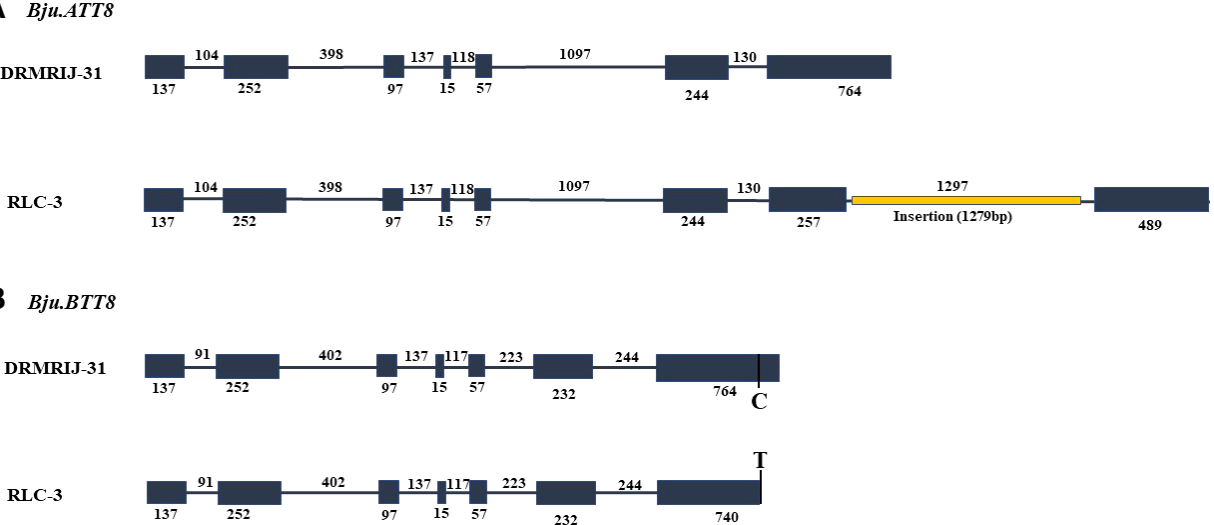
Gene structure of *Bju.TT8* homologs, exonic region represented by grey boxes and grey line denoting the intronic region **(A)** Diagrammatic representation of Gene differences observed in DRMRIJ-31 and RLC-3 for *Bju.ATT8* gene, RLC-3 had large insertion of 1279 bp (depicted by orange line) in the seventh exon **(B)** Gene differences observed in DRMIJ-31 and RLC-3 for *Bju.BTT8* gene, RLC-3 had a SNP (C→T) in the seventh exon.

The *Bju.TT8* homologs amplified using respective primers were named as *Bju.ATT8 and Bju.BTT8*, referring to their location at chromosome A09 and B03, respectively. The *in-silico* sequence analysis showed that *Bju.TT8* genes were consisting of 7 exons and 6 introns. The length of the first five exons of *Bju.TT8* homologs were well conserved; however, 6^th^ and 7^th^ exons showed variation in the sequence length (**Figure 2**). *Bju.TT8* homolog sequences when compared between two parents, DRMRIJ-31 and RLC-3, showed that RLC-3 has long insertion of 1279 bp in the 7^th^ exon (from 3047bp to 4325bp) of *Bju.ATT8* gene resulting an additional intron of 1297 bp and a single point mutation (C/T) was observed at position 2742bp from start point, in the 7^th^ exon of *Bju.BTT8* gene (**Figure 2**).

### Development of functional markers for *Bju.ATT8* and *Bju.BTT8* genes

**Figure 3:**
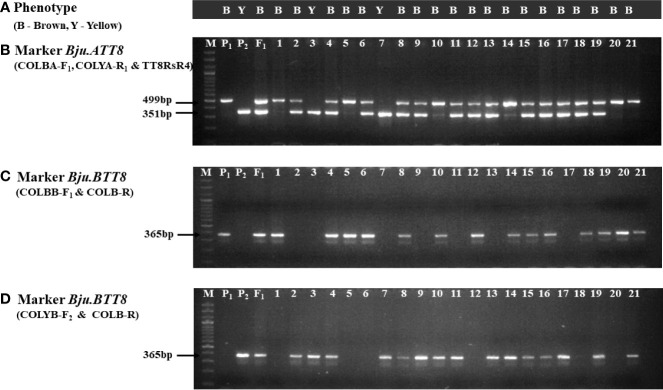
Markers segregation in F_2_ population **(A)** Phenotype of individual F_2_ plants (brown and yellow); **(B)** Gel image of *Bju.ATT8* marker; **(C, D)** Gel image of *Bju.BTT8* markers using two different combinations of forward primers; M-Ladder, P_1_-DRMRIJ-31, P_2_-RLC-3, F_1_-Hybrid, 1-21-F_2_ plants.

Located on A09 chromosome, *Bju.ATT8* gene had an insertion rendering it non-functional in RLC-3. Exploiting this insertion of 1279 bp in the RLC-3, we have developed a functional sequence-tagged sites (STS) marker, that comprised of a forward primer, COLBA-F_1,_ designed outside the insertion site (88bp from the insertion site), and the reverse primer TT8RsR4, from an earlier study (Padmaja et al., 2014) were developed. The amplified product of this primer set was 499bp in DRMRIJ-31 (brown seeded) and 1778 bp in RLC-3 (yellow seeded). However, the larger fragment of 1778 bp in RLC-3 seemed less user friendly due to its large size. Hence, an additional reverse primer, COLYA-R_1_ was designed using the sequences within the insertion (263bp inside the insertion site) to specifically target the yellow seeded parent. When put together in a multiplex PCR, COLBA-F_1_ and COLYA-R_1_ could amplify a 351bp fragment in RLC-3, while a 499 bp fragment was generated in DRMRIJ-31 with the help of the reverse primer, TT8RsR4 (**Figures 3A, B**; **Supplementary Figure 2A**). Interestingly, a large-sized amplicon (1778 bp) in RLC-3 generated by TT8RsR4 could be suppressed by changing the PCR conditions *i.e*., annealing temperature was kept at 62°C for 30s and extended for one minute at 72°C. The primer concentration of COLBA-F_1_, COLYA-R_1_, and TT8RsR4 were kept 10, 5 and 5 pM, respectively (**Table 2A**).

**Table 2:** Details of primers used for the development of functional markers for *Bju.TT8* genes.

SN	Gene	Primer	Primer Sequence (5’-3’)	Location	Annealing temperature and Annealing time	Extension Temperature and Extension time	Amplicon size	Polymorphism
A.	*Bju.ATT8*	COLBA-F_1_	GCAATATCAGCGAGTGGAGAA	A09	62^0^C, 30s	72^0^C, 1min	499 bp in DRMRIJ-31 (brown seeded) and 351 bp band in RLC-3 (yellow seeded)	Length
		COLYA-R_1_	ACAATTGGTGCCTCCTGTAAG					
		TT8RsR4	CTCAAAATGATTGTCGTTCAAGGCA					
B.	*Bju.BTT8*	COLBB-F_1_	GAGGTTAAAATAGCCATCCGTC	B03	60^0^C, 20s	72^0^C, 30s	365 bp band amplified only in DRMRIJ-31 (brown seeded)	Presence/ Absence
		COLB-R	GGGACATATATAGTGTTAGGTGAC					
		COLYB-F_2_	GAGGTTAAAATAGCCATCCGTT	B03	64^0^C, 20s	72^0^C, 30s	365 bp band amplified only in RLC-3 (yellow seeded)	Presence/ Absence
		COLB-R	GGGACATATATAGTGTTAGGTGAC					

The functional markers developed for *Bju.BTT8* gene located on B03 chromosome targeted the SNP (C/T) present in the seventh exon. Two forward (COLBB-F_1_, COLYB-F_2_) and one reverse (COLB-R) primers were developed. COLBB-F_1_ targeted the SNP nucleotide ‘C’ with an additional mismatch of ‘G’ at the third nucleotide position from the SNP at the 3’ end as suggested by Liu et al. (2012). COLYB-F_1_ was similar to COLBB-F_1_, but targeted the SNP nucleotide ‘T’. Although both the primers amplified a band size of 365bp, allele specificity of these markers were remarkable, since COLBB-F_1_ could amplify only the brown allele, while COLYB-F1 amplified the yellow allele. However, two separate PCR reactions having different primer combinations, COLBB-F_1_+COLB-R and COLYB-F_2_ + COLB-R were required to differentiate homozygotes and heterozygotes, because of their dominant nature (**Figures 3A, C, D**, **Supplementary Figure 2B**). Further, the PCR reaction for COLBB-F_1_ + COLB-R required annealing for the 20s at 60°C and extension at 72°C for 30s, while COLYB-F_2_ + COLB-R required annealing at 64°C for 20s and extension at 72°C for 30s (**Table 2B**). The concentration of forward and reverse primers was kept 10 pM each for PCR reaction.

### Validation of markers in F_2_ population

Functional markers validated over a F_2_ population of 526 plants, derived from the cross DRMRIJ-31/RLC3, the *Bju.ATT8* marker revealed a segregation of 129 AA, 269 Aa and 128 aa plants. ‘A’ represented the brown allele from DRMRIJ-31 and ‘a’ represented the yellow allele from RLC3. The phenotype of the F_2_ plants perfectly associated to the allelic status. Based on the goodness of fit, the marker segregation followed the expected ratio of 1:2:1 with a probability of 0.87. Similarly, the segregation of the *Bju.BTT8* gene has shown 133 BB, 247 Bb and 146 bb plants, having a probability of 0.27 (**Supplementary Figures 1B, C**, **Tables 1B, C**).

### Combined markers segregation

By combined marker analysis, 526 F_2_ plants were categorized into 9 different genotypic classes based on the marker allelic pattern as AABB (34), AABb (61), AAbb (34), AaBB (61), AaBb (122), Aabb (86), aaBB (38), aaBb (64) and aabb (26). The segregation of both markers was found to follow the expected genotypic ratio for mendelian dihybrids (1:2:1:2:4:2:1:2:1) with a probability of 0.27 (**Table 1D**). When F_2_ individual plants were categorized based on the presence of dominant alleles on either or both the loci, there were 34 plants had all four dominant alleles (AABB) identical to DRMRIJ-31. 122 plants had three dominant alleles (AABb or AaBB), 194 plant with two dominant alleles and 150 plants with one dominant allele. All the plants that carried dominant allele were having brown seed coat. Altogether, 26 plants carried all recessive alleles (aabb) identical to RLC-3 and having yellow seed coat colour (**Supplementary Figure 1D**).

Since both the genes (*Bju.ATT8* and *Bju.BTT8*) assorted independently and showed duplicate type gene action, a separate goodness of fit test was run for each marker to confirm the significance of their influence on the seed coat colour. Based on the allelic pattern, the segregation of *Bju.ATT8* and *Bju.BTT8 gene* showed perfect agreement with the expected pattern having a *χ^2^*-value of 1.73 and 7.06 having a probability of 0.63 and 0.07, respectively (**Tables 1E, F**).

### Validation of *Bju.TT8* allelic combinations among the mustard genotypes

Out of 130 genotypes used for validation, 96, 10, 9, and 12 *B. juncea* genotypes were having the genetic constitution of AABB, AAbb, aaBB, and aabb, respectively. Genotypes of the first three genotypic classes (AABB, AAbb, aaBB) had brown seed coat colour, whereas the fourth class (aabb) had yellow seed coat colour (**Table 3**). Of the remaining, contrary to the expectations, both the yellow and brown seeded genotypes of *B. rapa* produced wild type (brown) allele (AA; 499bp) with *Bju.ATT8* gene. As expected, there was no amplification of *Bju.BTT8* gene in *B. rapa*. Similarly, *Bju.ATT8* gene was not amplified in *B. nigra* genotype IC-281862; but *Bju.BTT8* did amplify with its wild type (brown) allele (BB; 365bp) (**Table 3**).

**Table 3:** Classification of mustard genotypes based on seed coat colour and their genotypic classes using *Bju.ATT8* (AA/aa) and *Bju.BTT8* (BB/bb) markers.

S.N.	Seed coat colour	Genotypic classes	Genotypes
1.	Brown	AABB	1. RE-8, 2. RE-13, 3. RE-14, 4. RE-35-4, 5. CN-101849, 6. CN-105308, 7. CN-101846, 8. CN-112920, 9. CN-105310, 10. CN-101834, 11. CN-34008, 12. CN-105313, 13. CN-105234, 14. CN-105311, 15. CN-101887, 16. DRMRIJ-17-46, 17. DJ-33, 18. DJ-86, 19. DTM-4, 20. DTM-12, 21. DTM-25, 22. DTM-50, 23. IM-3, 24. IM-17, 25. IM-22, 26. IM-59, 27. IM-66, 28. RE-15, 29. RE-11, 30. PR-2001-42, 31. PBR-97, 32. PCR-9403, 33. CN-105257, 34. CN-105312, 35. CN-105309, 36. RGN-73, 37. IM-76, 38. IM-39, 39. IC-597869, 40. IC-597873, 41. IM-170, 42. CN-101813, 43. CN-101845, 44. TN-3, 45. LES-42, 46. PM 30, 47. ELM-123, 48. RH-801, 49. AGRANI, 50. NPJ-246, 51. PM 25, 52. NPJ-240, 53. NPJ-245, 54. NPJ-230, 55. PM 26, 56. NRCHB-101, 57. Pusa Mahak, 58. PM 28, 59. NPJ-203, 60. NPJ 161, 61. Pusa Tarak, 62. Kranti, 63. Laxmi, 64. Varuna, 65. Pusa Bahar, 66. RH-1499-30, 67. RH-1566, 68. RH-1706, 69. RH-1734, 70. RH-1735, 71. RH-1745, 72. RH-761, 73. NRCDR-2, 74. NPJ-181, 75. RH-749, 76. RH-725, 77. BPR-543-2, 78. KMR(E)-19-1, 79. DRMRCI 116, 80. PRE-17-2, 81. BAUM-09-12-1, 82. PRE-17-5, 83. RH-1999-42, 84. RH-1999-18, 85. DRMR-2017-21, 86. NPJ-214, 87. NPJ-176, 88. Pusa Jagannath, 89. Pusa Bold, 90. RC-371-1, 91. RC-132, 92. IC-597949, 93. IC-597904, 94. IC-597881, 95. RC-1270, 96. DRMRIJ-31
2.	Brown	AAbb	1. CN-105306, 2. DJ-109, 3. DTM-34, 4. IM-46, 5. ELM-132, 6. LET-18, 7. PM 27, 8. RB-50, 9. IC-597910, 10. RC-891-1
3.	Brown	aaBB	1. IM-97, 2. IM-110, 3. IM-108, 4. I-79 (M), 5. LES-54, 6. PDZ-6, 7. NPJ 210, 8. RHUR-2-1, 9. GR-325
4.	Yellow	aabb	1. DJ-26, 2. Pusa Karishma, 3. EC-597318, 4. RLC-2, 5. PDZ-1, 6. PDZ-3, 7. PDZ-11, 8. Heera, 9. BIO-YSR, 10. Donskaja, 11. BEC-144, 12. RLC-3
5.	Brown	AA	1. TL-15 *(B. rapa)*
6.	Yellow	AA	1. Pusa Gold (*B. rapa)*
7.	Brown	BB	1. IC-281862 (*B. nigra*)

### Comparative genomics of *TT8* sequences

Multiple sequence alignment of available and predicted *TT8* sequences of different *Brassica* species *i.e., B. rapa* (XM_009115326.3, HQ337791.1, GU255866.1)*, B. nigra* (cv. YZ12151, Sangam)*, B. oleracea* (GU255867.1, GU219990.1)*, B. napus* (NM_001315974.2, GU255864.1, GU255865.1, cv. Da-Ae)*, B. juncea* (KJ942581.1, cv. Varuna, DRMRIJ-31, RLC-3, Heera, AU-213, Sichuan Huangzi, T84-66)*, B. carinata* (cv. sxm20200214) with *A. thaliana* (NM_117050.3) showed >83% homology for coding sequences (CDS) and >77% homology for protein sequences across the *B. species* (**Supplementary Tables 1**, **2**). Comparison of *Bju.ATT8* and *Bju.BTT8* in DRMRIJ-31 showed 95.2% CDS similarity and 92.3% protein identity. These sequences were 100% similar to that of published sequences (Padmaja et al., 2014; Xie et al., 2014). Further, CDS and protein sequences of *Bju.ATT8* showed high similarity (99.5% for CDS and 99.42% for protein) with *TT8* gene of *B. rapa* (cv. Chiffu, Chiifu-401-42), whereas *Bju.BTT8* showed high sequence similarity (99.9% for CDS and 99.8% for protein) with *TT8* gene of *B. nigra* (cv. YZ12151, Sangam). In the case of RLC-3, the CDS for both the *Bju.TT8* homologs showed 95.2% similarity and 92% protein identity. These sequences were 100% similar to that of Heera, a yellow seeded cultivar (Padmaja et al., 2014). Similarly, the *TT8* homologs of other two tetraploid species *i.e., B. napus* and *B. carinata* had shown greater homology with their progenitors at CDS as well as protein level (**Supplementary Tables 1**, **2**). In general, the CDS region of *TT8* comprised of at least 1530 bp that codes for protein of 509 amino acids. However, number of base pairs and amino acids for *TT8* gene varied across the *B. species* with a range of CDS sequence of 1530bp to 1566bp and protein sequence of 509 amino acid to 521 amino acids (**Supplementary Table 3**). Interestingly, CDS and amino acid length of *TT8* in different cultivars within a diploid species was identical, whereas the length varies among the species. *B. rapa* (cv. Chiffu, Chiifu-401-42, Tsuda), *B. nigra* (cv. YZ12151, Sangam), *B. oleracea* (cv. C10, Stovepipe) have 1566, 1554, 1551 bp CDS and 521, 517, 516 amino acids, respectively. However, in *B. juncea* differences were observed in CDS and amino acid length of *TT8* homologs among different cultivars. The black seeded cultivars (Tumida, DRMRIJ-31, Varuna, T84-66) have 1566 bp CDS and 521 amino acid sequence for *Bju.ATT8* gene, while yellow seeded cultivars (RLC-3, Heera, AU-213) have 1548 bp CDS and 515 amino acids. Likewise, for *Bju.BTT8* gene, black seeded cultivars have 1554 bp CDS and 517 amino acids, but yellow seeded cultivars have 1548 bp CDS and 515 amino acids. In addition, other tetraploid brassicas *i.e.*, *B. napus* and *B. carinata*, have CDS and amino acid length of *TT8* homologs identical to their respective diploid genomes (**Supplementary Table 3**).

## Discussion

Brown seed coat colour in *Brassicas* is a result of proanthocyanidin accumulation in the inner integuments of seed coat (Lepiniec et al., 2006). Working out the genetics of this trait, several previous studies have suggested inheritance pattern ranging from monogenic to multigenic in species of *Brassica* triangle. Although a variation in the seed coat colour intensity was also observed where brown seeded plants had more variation than yellow seeded plants, but these seeds were broadly classified into two categories *i.e.*, brown and yellow. Further, a difference in seed coat colour phenotyping of direct and reciprocal F_1_ crosses was also reported suggesting the trait is influenced by maternal effect. McGugan (1948) while describing the botany of *Brassica* genus observes that seed coat tissues contain the inner integument (pigment layer) that provides the colour to the seeds. Since the pigment layer of the seed coat is developed from integuments of the ovule, coat colour shows the maternal effect (Lepiniec et al., 2006), and therefore shows segregation only in F_2_ sporophyte. Also, the colour of F_1_ seeds is identical to the seeds of the female parent, irrespective of the nature of nuclear genes governing the trait (Padmaja et al., 2005). The first available report of inheritance of seed coat colour in *B. juncea*, suggests involvement of two duplicate genes with purple/brown being dominant over yellow (Sun, 1945). Several later studies, also confirmed a duplicate dominant type gene interaction, wherein the presence of a single dominant allele of any gene would be sufficient enough to produce brown seed coat colour, whereas individuals having all the recessive alleles will produce yellow seed coat colour (Vera et al., 1979; Vera and Woods, 1982; Padmaja et al., 2005; Yan et al., 2009). However, monogenic inheritance was also reported (Li et al., 2012; Ren et al., 2017) including in *B. juncea* (2016; Xu et al., 2010; Huang et al., 2012). Under a digenic dominant system with duplicate effect, like that is observed in this study, monogenic inheritance is possible when the brown seeded parent is having single homozygous dominant gene (either AA or BB) at one locus and the other gene in homozygous recessive state. In this study too, we have observed that some of the brown seeded genotypes having *AAbb* and *aaBB* genetic constitution for seed coat colour genes, supported the monogenic inheritance. The variation in seed coat colour intensity noticed among the brown seeded types was later attributed to prevailing environmental conditions (Deynze et al., 1993), and/or genetic factors such as minor genes/QTLs (Xu et al., 2010). In support of this, recently, a minor QTL, *qSC3.1* with two major QTLs, *qSC9.1 and qSCb9.1* controlling the seed coat colour was reported in the RILs and successfully validated in CSSLs of *B. rapa* (Zhang et al., 2019).

Flavonoid metabolism is an integral process in the seed coat pigmentation in *Brassica* seeds through proanthocyanidin accumulation in the inner integuments (Shirley, 1998; Lepiniec et al., 2006). Several mutants collectively known as *transparent testa (tt)* (Koornneef, 1990) identified in the cruciferous model species, *Arabidopsis thaliana* and there are 11 genes reported (Shirley et al., 1995). Most important among these genes, such as *TT2*, *TT8, TTG1* and *TTG2* encoded transcription factors that regulated the flavonoid metabolism in developing siliquae (Coen et al., 2020). Located on different genomic regions across *Brassica* species, these loci drive failed accumulation of proanthocyanidin (PA), leading to pigment less layer, resulting in yellow seeds. However, different *TT* loci are found to be involved in the different molecular and biochemical mechanisms (Baudry et al., 2004; Lepiniec et al., 2006; Chai et al., 2009). *TT1* gene was found to positively regulate the PA biosynthetic genes (Appelhagen et al., 2011), whereas, the *TT8* gene was found to have a role in seed coat colour development (Li et al., 2012; Padmaja et al., 2014; Zhai et al., 2020).

Investigated in this study, using two contrasting *B. juncea* cultivars, DRMRIJ-31 (brown seeded) and RLC-3 (yellow seeded), the nucleotide sequence of the *Bju.TT8* genes revealed two homologs produced similar results as earlier reported by Padmaja et al. (2014), using two other cultivars, Varuna (brown seeded) and Heera (yellow seeded). Their corresponding mutant alleles, *Bju.att8* showed an insertion of 1279 bp in the seventh exon while the *Bju.btt8* allele showed a C/T substitution on the seventh exon leading to a stop codon, TAA (Padmaja et al., 2014). Both the mutations yielded aberrant non-functional proteins, leading to yellow seeded phenotype. In this study, having found similar alleles, across a set of 127 *B. juncea* germplasm lines, we conclude that the coat colour variation in Indian mustard is governed by two duplicate genes, *Bju.ATT8* and *Bju.BTT8*, possibly indicating a common source of origin.

Padmaja et al. (2014) have developed primers flanking *Bju.ATT8* and *Bju.BTT8* to amplify the entire genes and suggested that the polymorphism in these genes could be used in marker assisted crop improvement. Accordingly, targeting the insertion site in the mutant gene (*Bju.att8*), we have developed an STS marker which amplified a 499 bp fragment with the wild type allele (*Bju.ATT8*), while it amplified a very long fragment (1.78kb) in the yellow seeded mutant. Although the absence of 499 bp allele can indicate the presence of the mutant allele, we have designed an additional marker to target an intermediate region of the insertion site, that could amplify a specific band of 351 bp in the mutant alone for confirming the presence of the *Bju.att8* allele. The amplified products of this primer set (499bp in brown seeded and 351bp in yellow seeded parent) were more user convenient than that of Padmaja et al. (2014) (3550 bp in brown seeded parent and 4708 bp in the yellow seeded parent). This multiplex marker system can be used for selecting the target allele for the *Bju.ATT8*. In earlier studies also, a similar approach was used for developing markers for *BjuBCYP79F1* gene-regulating synthesis of aliphatic glucosinolates in *B. juncea* (Sharma et al., 2016). However, the use of these marker may be limited to *B. juncea*, because a yellow seeded genotype of *B. rapa* (Pusa Gold) failed to produce the desired amplicon of 351 bp, and instead produced the 499 bp allele typical to that of the wild type allele. This indicates that a different mutation is the cause of the yellow allele in *B. rapa* cv. Pusa Gold. Two different and independent insertional mutations in the *TT8* gene were earlier reported in *B. rapa (BrTT8*; Li et al., 2012) and *B. juncea (Bju.ATT8;* Padmaja et al., 2014) that cause yellow seed coat. It evidently suggests that insertional event in *Bju.ATT8* gene of *B. juncea* had occurred after its evolution from *B. rapa*.

Targeting the *Bju.BTT8* gene for marker-based selection was a challenge, because a SNP was responsible for the mutation. Moreover, *Bju.BTT8* gene was found to be more conserved than the *Bju.ATT8* gene. The wild type nucleotide in the SNP was ‘C’ that produced the brown seeded plants, which when changed to T produced the yellow seeded plants. Although SNPs are the most common type of DNA polymorphism (Brookes, 1999), the sequencing techniques targeting a specific SNP in molecular breeding programs is still a cumbersome process. Development of markers targeting functional polymorphism in the form of PCR based markers therefore still remains the choice of the breeders. There are several methods in use to target an SNP in marker development, such as the use of cleaved amplified polymorphic sequence (CAPS), derived CAPS (dCAPS) and allele specific primers. Allele-specific PCR based markers are cost-effective and require less operational time, the use of CAPS and dCAPS markers depends on restriction sites by endonuclease and are expensive (Liu et al., 2012). However, precision of the allele specific markers depends on the PCR conditions. We have developed two separate allele specific primers for *Bju.BTT8* gene, one targeting ‘C’ nucleotide and the other targeting ‘T’. A common reverse primer was used. To improve the allele specificity a mismatch was added at the third nucleotide from the SNP at the 3’ end of the primer (Liu et al., 2012). Under the PCR conditions described in this study, both the primers could perfectly differentiate both the alleles producing an amplicon of 365 bp. Two independent PCRs with independent resolution of amplicons in separate electrophoretic gels were required to test the allele specificity and a comparative evaluation of both the gels were required to identify the heterozygotes.

Linked markers for seed coat colour in *B. juncea* were reported in several studies, such as AFLP markers (Negi et al., 2000), three QTLs *viz. SC-B04*, *SC-A10*, and *SC-A06* (Mahmood et al., 2005), gene linked markers *Na10-A08* and *Ni4-F11* (Padmaja et al., 2005), IP and SCAR markers (Huang et al., 2016). However, none of these linked markers were prove to be effective for marker assisted selection. The functional markers (Gupta and Rustgi, 2004) developed in this study targeting *Bju.TT8* homologs showed unambiguous Mendelian segregation with no recombination between marker and trait, assuring their high reliability in marker-assisted breeding. Efforts to develop gene-based and functional markers in *Brassica* species, have been made in the past to select *FAE* genes governing erucic acid content (Saini et al., 2016, Saini et al., 2019), seed coat colour (Padmaja et al., 2014), erucic acid genes (Gill et al., 2021), and *BnFAD2* gene controlling high oleic acid content (Fu et al., 2021).

Validation of the newly developed markers in a panel of 127 genotypes of *B. juncea* proved their perfect utility in selection, particularly in *B. juncea*, which could differentiate the population into four classes based on allelic combinations. All these combinations, interestingly, did not deviate from the expected seed coat colour. Saini et al. (2016) have conducted a similar study to categorize different genotypes of *B. juncea* based on allelic combinations of *FAE* genes. As explained earlier, one exception we noticed pertains to a *B. rapa* genotype, Pusa Gold. Recently, it was reported that a major QTL for yellow seed coat, *cqSC-A09* located on chromosome A09 (same chromosome as that of *Bju.ATT8)* increases the oil and fibre content of the seed in *B. napus* (Chao et al., 2022). If, a similar effect exists with *Bju.ATT8*, breeding *B. juncea* carrying this mutant allele can definitely have advantage towards Indian mustard improvement.

Comparison of homolog sequences of the *TT8* gene within and between the *Brassica* genomes indicates high level of conservation, suggesting a descent from a common progenitor during evolution. Similar observations were also made for *TT12* gene (related again to seed coat colour) of different *Brassica* species (Chai et al., 2009). In the present study, consanguineous relationships indicated by the sequence homology among the *TT8* genes, suggest that in tetraploid *Brassicas* the homologs might have come from their respective diploid progenitors before the speciation, as more proximity was observed with the progenitor genes than among the homologs themselves present within the same species. Based on this hypothesis, it is apparent that *Bju.TT8* homologs descended directly from *B. rapa* and *B. nigra* and evolved subsequently and independently. The similar predictions about progenitors of seed coat colour genes in *B. juncea* were documented earlier (Negi et al., 2000).

In this study, we confirm that the duplicate dominant genes for seed coat colour in *B. juncea*, are the homologs of *TT8* gene originating from the two diploid progenitor species, *B. rapa* and *B. nigra*. Both the genes affect the nucellar pigmentation in the testa, thereby exhibiting maternal effect. They code for transcription factors affecting proanthocyanidin metabolism, and mutation in both the genes results in yellow seeds in place of wild type brown seeds. In Indian mustard, two major mutations exist in both the homologs, both affecting the seventh exon of the *TT8* gene resulting in yellow seed coat colour. These genes are the major genes that control seed coat colour inheritance; however, the role of environmental factors and minor genes could not be ignored. In the breeding perspective, there is a need to improve seed storability which is associated with brown seed, while higher oil and protein contents are attributed to yellow seed. Therefore, depending on the breeding objective, it is essential to have functional marker systems to target the desired choice of seed coat colour. We have developed functional markers in this study targeting both wild and mutant alleles, which have been validated in a segregating as well as a germplasm assembly. We conclude that these markers are highly reliable, cost-effective and could be used in marker-assisted breeding. The results from this study also indicate that the marker systems developed herein are for Indian mustard alone, as different genetic systems governing seed coat colour may be operational in other *Brassica* species.

## Data availability statement

The original contributions presented in the study are included in the article/**Supplementary Material**. Further inquiries can be directed to the corresponding author.

## Author contributions

Conceptualization- NS and DY. Investigation- MP and YT. Methodology- MP. Validation- RC and MP. Formal analysis- MP and KV. Resources- SV and NS. Software- MP and RC. Data curation- PP and JN. Writing- MP. Writing, review and editing- NS, KV, YT, SV and DY. Visualization- NS. Supervision- NS. All authors contributed to the article and approved the submitted version.

## Acknowledgments

The authors are grateful to Director, ICAR-IARI, New Delhi for providing fellowship to MP and CRP-MB, ICAR, New Delhi for providing required facility during research programme.

## Conflict of interest

The authors declare that the research was conducted in the absence of any commercial or financial relationships that could be construed as a potential conflict of interest.

## Publisher’s note

All claims expressed in this article are solely those of the authors and do not necessarily represent those of their affiliated organizations, or those of the publisher, the editors and the reviewers. Any product that may be evaluated in this article, or claim that may be made by its manufacturer, is not guaranteed or endorsed by the publisher.
