# Supplementary material for: Seed coat colour of Indian mustard [Brassica juncea (L.) Czern. and Coss.] is associated with Bju.TT8 homologs identifiable by targeted functional markers
Source: Front Plant Sci. 2022 Oct 5;13:1012368. doi: 10.3389/fpls.2022.1012368 (PMC9581272; doi:10.3389/fpls.2022.1012368)
Supplement: Supplementary file 1 [file DataSheet_1.docx]

Supplementary Material


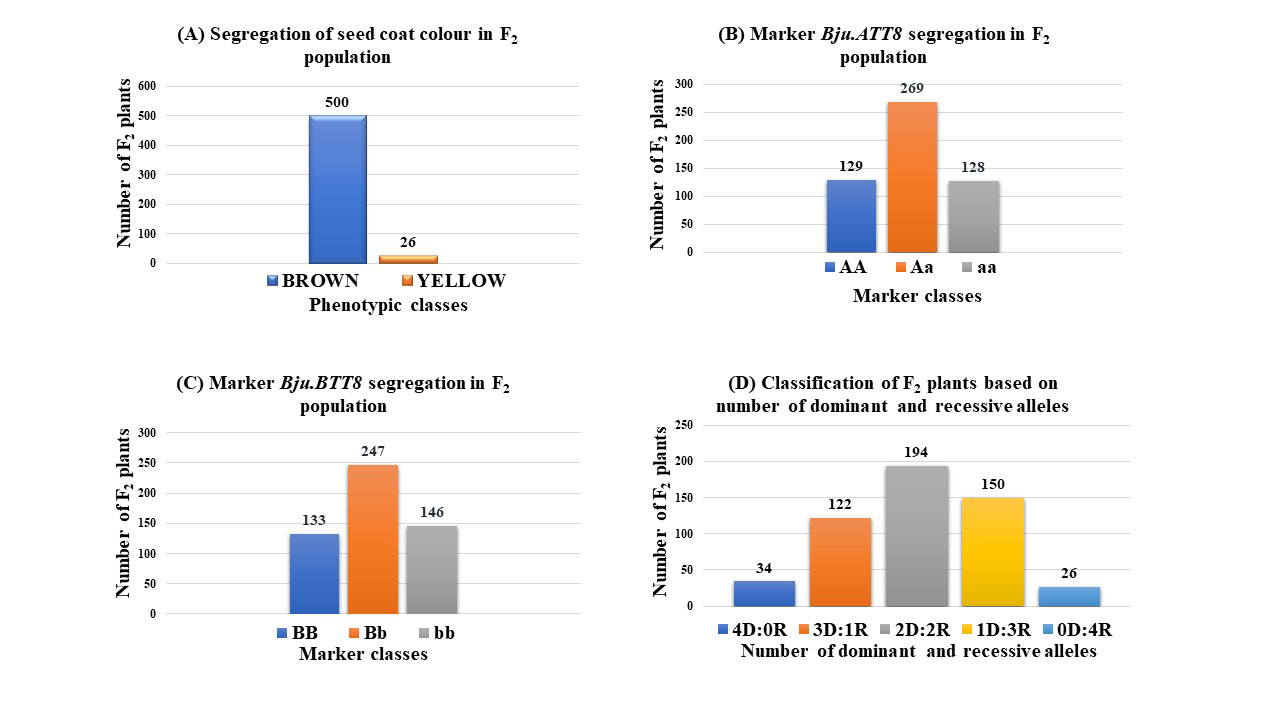


**SUPPLEMENTARY FIGURE 1 |** Graphical representation of genotypic and phenotypic segregation in F_2_ population. **(A)** Phenotypic segregation of seed coat colour in F_2_ population **(B)** *Bju.ATT8* marker segregation in F_2_ population **(C)** *Bju.BTT8* marker segregation in F_2_ population **(D)** Classification of F_2_ plants based on number of dominant and recessive alleles.


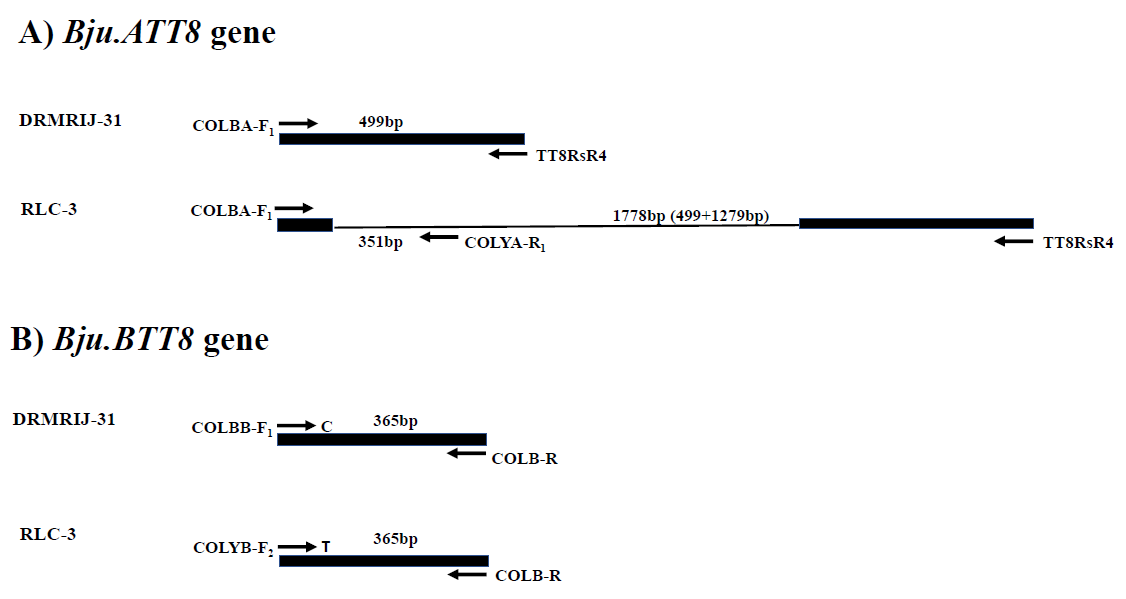


**SUPPLEMENTARY FIGURE 2 |** Diagrammatic illustration of different sets of primers (forward and reverse) designed for *Bju.TT8* homologs. **(A)** In *Bju.ATT8 gene*, primers COLA-F_1_ and TT8RsR4 were from outside the inserted region (denoted by thick line), whereas COLYA-R1 from inserted region (denoted by thin line). These primer sets amplified an amplicon of 499 bp in DRMRIJ-31 and 351 bp in RLC-3. (**B)** In *Bju.ATT8 gene*, allele specific primers COLBB-F_1_ and COLYB-F_2_ were designed using SNP ‘C’ and ‘T’ respectively, at the 3’ end. The Primer set, COLBB-F_1_ + COLB-R amplified an amplicon of 365 bp in DRMRIJ-31, whereas other primer set, COLYB-F_2_ + COLB-R amplified an amplicon of 365 bp in RLC-3.

**SUPPLEMENTARY TABLE 1 |** Coding sequence (CDS) similarity matrix of *TT8* gene sequences among *A. thaliana* and different *Brassica species*

| **SN** | **Particulars** | **1** | **2** | **3** | **4** | **5** | **6** | **7** | **8** | **9** | **10** | **11** | **12** | **13** | **14** | **15** | **16** | **17** | **18** | **19** | **20** | **21** | **22** | **23** | **24** | **25** | **26** | **27** | **28** | **29** |
| --- | --- | --- | --- | --- | --- | --- | --- | --- | --- | --- | --- | --- | --- | --- | --- | --- | --- | --- | --- | --- | --- | --- | --- | --- | --- | --- | --- | --- | --- | --- |
| 1 | *A. thaliana* (NM_117050.3) |  |  |  |  |  |  |  |  |  |  |  |  |  |  |  |  |  |  |  |  |  |  |  |  |  |  |  |  |  |
| 2 | *B. juncea* (RLC-3-B03) | 85.2 |  |  |  |  |  |  |  |  |  |  |  |  |  |  |  |  |  |  |  |  |  |  |  |  |  |  |  |  |
| 3 | *B. juncea* (Heera-B03) | 85.2 | 100 |  |  |  |  |  |  |  |  |  |  |  |  |  |  |  |  |  |  |  |  |  |  |  |  |  |  |  |
| 4 | *B. juncea* (AU-213-B08) | 85.2 | 100 | 100 |  |  |  |  |  |  |  |  |  |  |  |  |  |  |  |  |  |  |  |  |  |  |  |  |  |  |
| 5 | *B. juncea* (Sichuan Huangzi-B08) | 85.2 | 100 | 100 | 100 |  |  |  |  |  |  |  |  |  |  |  |  |  |  |  |  |  |  |  |  |  |  |  |  |  |
| 6 | *B. carinata* (sxm20200214-B1) | 84.5 | 99.9 | 99.9 | 99.9 | 99.9 |  |  |  |  |  |  |  |  |  |  |  |  |  |  |  |  |  |  |  |  |  |  |  |  |
| 7 | *B. juncea* (DRMRIJ-31-B03) | 84.5 | 99.9 | 99.9 | 99.9 | 99.9 | 99.9 |  |  |  |  |  |  |  |  |  |  |  |  |  |  |  |  |  |  |  |  |  |  |  |
| 8 | *B. juncea* (Varuna-B03) | 84.5 | 99.9 | 99.9 | 99.9 | 99.9 | 99.9 | 100 |  |  |  |  |  |  |  |  |  |  |  |  |  |  |  |  |  |  |  |  |  |  |
| 9 | *B. juncea* (T84-66-B08) | 84.5 | 99.9 | 99.9 | 99.9 | 99.9 | 99.9 | 100 | 100 |  |  |  |  |  |  |  |  |  |  |  |  |  |  |  |  |  |  |  |  |  |
| 10 | *B. nigra* (Sangam-B03) | 84.5 | 99.9 | 99.9 | 99.9 | 99.9 | 99.9 | 99.9 | 99.9 | 99.9 |  |  |  |  |  |  |  |  |  |  |  |  |  |  |  |  |  |  |  |  |
| 11 | *B. nigra* (YZ12151-B03) | 84.5 | 99.9 | 99.9 | 99.9 | 99.9 | 99.9 | 99.9 | 99.9 | 99.9 | 100 |  |  |  |  |  |  |  |  |  |  |  |  |  |  |  |  |  |  |  |
| 12 | *B. carinata* (sxm20200214-C4) | 83.4 | 95.2 | 95.2 | 95.2 | 95.2 | 95.1 | 95.1 | 95.1 | 95.1 | 95.1 | 95.1 |  |  |  |  |  |  |  |  |  |  |  |  |  |  |  |  |  |  |
| 13 | *B. oleracea* (GU255867.1) | 83.4 | 95.2 | 95.2 | 95.2 | 95.2 | 95.1 | 95.1 | 95.1 | 95.1 | 95.1 | 95.1 | 100 |  |  |  |  |  |  |  |  |  |  |  |  |  |  |  |  |  |
| 14 | *B. oleracea* (GU219990.1) | 83.7 | 95.4 | 95.4 | 95.4 | 95.4 | 95.3 | 95.3 | 95.3 | 95.3 | 95.3 | 95.3 | 99.5 | 99.5 |  |  |  |  |  |  |  |  |  |  |  |  |  |  |  |  |
| 15 | *B. napus* (GU255865.1) | 83.6 | 95.3 | 95.3 | 95.3 | 95.3 | 95.3 | 95.3 | 95.3 | 95.3 | 95.2 | 95.2 | 99.6 | 99.6 | 99.9 |  |  |  |  |  |  |  |  |  |  |  |  |  |  |  |
| 16 | *B. napus* (Da-Ae-C09) | 83.6 | 95.3 | 95.3 | 95.3 | 95.3 | 95.3 | 95.3 | 95.3 | 95.3 | 95.2 | 95.2 | 99.6 | 99.6 | 99.9 | 100 |  |  |  |  |  |  |  |  |  |  |  |  |  |  |
| 17 | *B. juncea* (RLC-3-A09) | 83.8 | 95.2 | 95.2 | 95.2 | 95.2 | 95.1 | 95.2 | 95.2 | 95.2 | 95.1 | 95.1 | 98 | 98 | 98 | 98.1 | 98.1 |  |  |  |  |  |  |  |  |  |  |  |  |  |
| 18 | *B. juncea* (Heera-A09) | 83.8 | 95.2 | 95.2 | 95.2 | 95.2 | 95.1 | 95.2 | 95.2 | 95.2 | 95.1 | 95.1 | 98 | 98 | 98 | 98.1 | 98.1 | 100 |  |  |  |  |  |  |  |  |  |  |  |  |
| 19 | *B. juncea* (AU-213-A09) | 83.6 | 95.2 | 95.2 | 95.2 | 95.2 | 95.1 | 95.1 | 95.1 | 95.1 | 95.1 | 95.1 | 98 | 98 | 98 | 98 | 98 | 99.8 | 99.8 |  |  |  |  |  |  |  |  |  |  |  |
| 20 | *B. juncea* (Sichuan Huangzi-A09) | 83.6 | 95.2 | 95.2 | 95.2 | 95.2 | 95.1 | 95.1 | 95.1 | 95.1 | 95.1 | 95.1 | 98 | 98 | 98 | 98 | 98 | 99.8 | 99.8 | 100 |  |  |  |  |  |  |  |  |  |  |
| 21 | *B. rapa* (GU255866.1) | 83.8 | 95.4 | 95.4 | 95.4 | 95.4 | 95.2 | 95.3 | 95.3 | 95.3 | 95.2 | 95.2 | 98.1 | 98.1 | 98.1 | 98.2 | 98.2 | 99.6 | 99.6 | 99.5 | 99.5 |  |  |  |  |  |  |  |  |  |
| 22 | *B. rapa* (XM_009115326.3 | 83.8 | 95.4 | 95.4 | 95.4 | 95.4 | 95.2 | 95.3 | 95.3 | 95.3 | 95.2 | 95.2 | 98.1 | 98.1 | 98.1 | 98.2 | 98.2 | 99.6 | 99.6 | 99.5 | 99.5 | 100 |  |  |  |  |  |  |  |  |
| 23 | *B. rapa* (HQ337791.1) | 83.9 | 95.4 | 95.4 | 95.4 | 95.4 | 95.3 | 95.4 | 95.4 | 95.4 | 95.3 | 95.3 | 98.2 | 98.2 | 98.2 | 98.3 | 98.3 | 99.9 | 99.9 | 99.8 | 99.8 | 99.7 | 99.7 |  |  |  |  |  |  |  |
| 24 | *B. napus* (GU255864.1) | 83.9 | 95.4 | 95.4 | 95.4 | 95.4 | 95.3 | 95.4 | 95.4 | 95.4 | 95.3 | 95.3 | 98.2 | 98.2 | 98.2 | 98.3 | 98.3 | 99.9 | 99.9 | 99.8 | 99.8 | 99.7 | 99.7 | 100 |  |  |  |  |  |  |
| 25 | *B. napus* (NM_001315974.2) | 83.9 | 95.4 | 95.4 | 95.4 | 95.4 | 95.3 | 95.4 | 95.4 | 95.4 | 95.3 | 95.3 | 98.2 | 98.2 | 98.2 | 98.3 | 98.3 | 99.9 | 99.9 | 99.8 | 99.8 | 99.7 | 99.7 | 100 | 100 |  |  |  |  |  |
| 26 | *B. napus* (Da-Ae-A09) | 83.9 | 95.4 | 95.4 | 95.4 | 95.4 | 95.3 | 95.4 | 95.4 | 95.4 | 95.3 | 95.3 | 98.2 | 98.2 | 98.2 | 98.3 | 98.3 | 99.9 | 99.9 | 99.8 | 99.8 | 99.7 | 99.7 | 100 | 100 | 100 |  |  |  |  |
| 27 | *B. juncea* (KJ942581.1) | 83.8 | 95.2 | 95.2 | 95.2 | 95.2 | 95.1 | 95.2 | 95.2 | 95.2 | 95.1 | 95.1 | 98 | 98 | 98 | 98.1 | 98.1 | 99.8 | 99.8 | 100 | 100 | 99.5 | 99.5 | 99.8 | 99.8 | 99.8 | 99.8 |  |  |  |
| 28 | *B. juncea* (DRMRIJ-31-A09) | 83.8 | 95.2 | 95.2 | 95.2 | 95.2 | 95.1 | 95.2 | 95.2 | 95.2 | 95.1 | 95.1 | 98 | 98 | 98 | 98.1 | 98.1 | 99.8 | 99.8 | 100 | 100 | 99.5 | 99.5 | 99.8 | 99.8 | 99.8 | 99.8 | 100 |  |  |
| 29 | *B. juncea* (Varuna-A09) | 83.8 | 95.2 | 95.2 | 95.2 | 95.2 | 95.1 | 95.2 | 95.2 | 95.2 | 95.1 | 95.1 | 98 | 98 | 98 | 98.1 | 98.1 | 99.8 | 99.8 | 100 | 100 | 99.5 | 99.5 | 99.8 | 99.8 | 99.8 | 99.8 | 100 | 100 |  |
| 30 | *B. juncea* (T84-66-A09) | 83.8 | 95.2 | 95.2 | 95.2 | 95.2 | 95.1 | 95.2 | 95.2 | 95.2 | 95.1 | 95.1 | 98 | 98 | 98 | 98.1 | 98.1 | 99.8 | 99.8 | 100 | 100 | 99.5 | 99.5 | 99.8 | 99.8 | 99.8 | 99.8 | 100 | 100 | 100 |

**SUPPLEMENTARY TABLE 2 |** Protein similarity matrix of *TT8* gene sequences among *A. thaliana* and different *Brassica* *species*

| **SN** | **Particulars** | **1** | **2** | **3** | **4** | **5** | **6** | **7** | **8** | **9** | **10** | **11** | **12** | **13** | **14** | **15** | **16** | **17** | **18** | **19** | **20** | **21** | **22** | **23** | **24** | **25** | **26** | **27** | **28** | **29** |
| --- | --- | --- | --- | --- | --- | --- | --- | --- | --- | --- | --- | --- | --- | --- | --- | --- | --- | --- | --- | --- | --- | --- | --- | --- | --- | --- | --- | --- | --- | --- |
| 1 | *A. thaliana* (NM_117050.3) |  |  |  |  |  |  |  |  |  |  |  |  |  |  |  |  |  |  |  |  |  |  |  |  |  |  |  |  |  |
| 2 | *B. juncea* (DRMRIJ-31-B03) | 78.9 |  |  |  |  |  |  |  |  |  |  |  |  |  |  |  |  |  |  |  |  |  |  |  |  |  |  |  |  |
| 3 | *B. juncea* (RLC-3-B03) | 79.8 | 100 |  |  |  |  |  |  |  |  |  |  |  |  |  |  |  |  |  |  |  |  |  |  |  |  |  |  |  |
| 4 | *B. juncea* (Varuna-B03) | 78.9 | 100 | 100 |  |  |  |  |  |  |  |  |  |  |  |  |  |  |  |  |  |  |  |  |  |  |  |  |  |  |
| 5 | *B. juncea* (Heera-B03) | 79.8 | 100 | 100 | 100 |  |  |  |  |  |  |  |  |  |  |  |  |  |  |  |  |  |  |  |  |  |  |  |  |  |
| 6 | *B. juncea* (AU-213-B08) | 79.8 | 100 | 100 | 100 | 100 |  |  |  |  |  |  |  |  |  |  |  |  |  |  |  |  |  |  |  |  |  |  |  |  |
| 7 | *B. juncea* (Sichuan Huangzi-B08) | 79.8 | 100 | 100 | 100 | 100 | 100 |  |  |  |  |  |  |  |  |  |  |  |  |  |  |  |  |  |  |  |  |  |  |  |
| 8 | *B. juncea* (T84-66-B08) | 78.9 | 100 | 100 | 100 | 100 | 100 | 100 |  |  |  |  |  |  |  |  |  |  |  |  |  |  |  |  |  |  |  |  |  |  |
| 9 | *B. carinata* (sxm20200214-B1) | 78.9 | 100 | 100 | 100 | 100 | 100 | 100 | 100 |  |  |  |  |  |  |  |  |  |  |  |  |  |  |  |  |  |  |  |  |  |
| 10 | *B. nigra* (Sangam-B03) | 78.7 | 99.8 | 99.8 | 99.8 | 99.8 | 99.8 | 99.8 | 99.8 | 99.8 |  |  |  |  |  |  |  |  |  |  |  |  |  |  |  |  |  |  |  |  |
| 11 | *B. nigra* (YZ12151-B03) | 78.7 | 99.8 | 99.8 | 99.8 | 99.8 | 99.8 | 99.8 | 99.8 | 99.8 | 100 |  |  |  |  |  |  |  |  |  |  |  |  |  |  |  |  |  |  |  |
| 12 | *B. juncea* (KJ942581.1) | 78.3 | 92.3 | 92.5 | 92.3 | 92.5 | 92.5 | 92.5 | 92.3 | 92.3 | 92.1 | 92.1 |  |  |  |  |  |  |  |  |  |  |  |  |  |  |  |  |  |  |
| 13 | *B. juncea* (DRMRIJ-31-A09) | 78.3 | 92.3 | 92.5 | 92.3 | 92.5 | 92.5 | 92.5 | 92.3 | 92.3 | 92.1 | 92.1 | 100 |  |  |  |  |  |  |  |  |  |  |  |  |  |  |  |  |  |
| 14 | *B. juncea* (Varuna-A09) | 78.3 | 92.3 | 92.5 | 92.3 | 92.5 | 92.5 | 92.5 | 92.3 | 92.3 | 92.1 | 92.1 | 100 | 100 |  |  |  |  |  |  |  |  |  |  |  |  |  |  |  |  |
| 15 | *B. juncea* (T84-66-A09) | 78.3 | 92.3 | 92.5 | 92.3 | 92.5 | 92.5 | 92.5 | 92.3 | 92.3 | 92.1 | 92.1 | 100 | 100 | 100 |  |  |  |  |  |  |  |  |  |  |  |  |  |  |  |
| 16 | *B. rapa* (GU255866.1) | 78.3 | 92.3 | 92.5 | 92.3 | 92.5 | 92.5 | 92.5 | 92.3 | 92.3 | 92.1 | 92.1 | 99.4 | 99.4 | 99.4 | 99.4 |  |  |  |  |  |  |  |  |  |  |  |  |  |  |
| 17 | *B. rapa* (XM_009115326.3 | 78.3 | 92.3 | 92.5 | 92.3 | 92.5 | 92.5 | 92.5 | 92.3 | 92.3 | 92.1 | 92.1 | 99.4 | 99.4 | 99.4 | 99.4 | 100 |  |  |  |  |  |  |  |  |  |  |  |  |  |
| 18 | *B. rapa* (HQ337791.1) | 78.5 | 92.5 | 92.7 | 92.5 | 92.7 | 92.7 | 92.7 | 92.5 | 92.5 | 92.3 | 92.3 | 99.6 | 99.6 | 99.6 | 99.6 | 99.8 | 99.8 |  |  |  |  |  |  |  |  |  |  |  |  |
| 19 | *B. napus* (GU255864.1) | 78.5 | 92.5 | 92.7 | 92.5 | 92.7 | 92.7 | 92.7 | 92.5 | 92.5 | 92.3 | 92.3 | 99.6 | 99.6 | 99.6 | 99.6 | 99.8 | 99.8 | 100 |  |  |  |  |  |  |  |  |  |  |  |
| 20 | *B. napus* (NM_001315974.2) | 78.5 | 92.5 | 92.7 | 92.5 | 92.7 | 92.7 | 92.7 | 92.5 | 92.5 | 92.3 | 92.3 | 99.6 | 99.6 | 99.6 | 99.6 | 99.8 | 99.8 | 100 | 100 |  |  |  |  |  |  |  |  |  |  |
| 21 | *B. napus* (Da-Ae-A09) | 78.5 | 92.5 | 92.7 | 92.5 | 92.7 | 92.7 | 92.7 | 92.5 | 92.5 | 92.3 | 92.3 | 99.6 | 99.6 | 99.6 | 99.6 | 99.8 | 99.8 | 100 | 100 | 100 |  |  |  |  |  |  |  |  |  |
| 22 | *B. juncea* (RLC-3-A09) | 78.2 | 92 | 92.3 | 92 | 92.3 | 92.3 | 92.3 | 92 | 92 | 91.8 | 91.8 | 99.6 | 99.6 | 99.6 | 99.6 | 99.4 | 99.4 | 99.6 | 99.6 | 99.6 | 99.6 |  |  |  |  |  |  |  |  |
| 23 | *B. juncea* (Heera-A09) | 78.2 | 92 | 92.3 | 92 | 92.3 | 92.3 | 92.3 | 92 | 92 | 91.8 | 91.8 | 99.6 | 99.6 | 99.6 | 99.6 | 99.4 | 99.4 | 99.6 | 99.6 | 99.6 | 99.6 | 100 |  |  |  |  |  |  |  |
| 24 | *B. juncea* (AU-213-A09) | 78.2 | 92.2 | 92.5 | 92.2 | 92.5 | 92.5 | 92.5 | 92.2 | 92.2 | 92 | 92 | 100 | 100 | 100 | 100 | 99.4 | 99.4 | 99.6 | 99.6 | 99.6 | 99.6 | 99.6 | 99.6 |  |  |  |  |  |  |
| 25 | *B. juncea* (Sichuan Huangzi-A09) | 78.2 | 92.2 | 92.5 | 92.2 | 92.5 | 92.5 | 92.5 | 92.2 | 92.2 | 92 | 92 | 100 | 100 | 100 | 100 | 99.4 | 99.4 | 99.6 | 99.6 | 99.6 | 99.6 | 99.6 | 99.6 | 100 |  |  |  |  |  |
| 26 | *B. carinata* (sxm20200214-C4) | 77.3 | 91.4 | 91.7 | 91.4 | 91.7 | 91.7 | 91.7 | 91.4 | 91.4 | 91.2 | 91.2 | 97.5 | 97.5 | 97.5 | 97.5 | 97.7 | 97.7 | 97.9 | 97.9 | 97.9 | 97.9 | 97.5 | 97.5 | 97.5 | 97.5 |  |  |  |  |
| 27 | *B. oleracea* (GU255867.1) | 77.3 | 91.4 | 91.7 | 91.4 | 91.7 | 91.7 | 91.7 | 91.4 | 91.4 | 91.2 | 91.2 | 97.5 | 97.5 | 97.5 | 97.5 | 97.7 | 97.7 | 97.9 | 97.9 | 97.9 | 97.9 | 97.5 | 97.5 | 97.5 | 97.5 | 100 |  |  |  |
| 28 | *B. oleracea* (GU219990.1) | 78.1 | 92.2 | 92.5 | 92.2 | 92.5 | 92.5 | 92.5 | 92.2 | 92.2 | 92 | 92 | 97.7 | 97.7 | 97.7 | 97.7 | 97.9 | 97.9 | 98.1 | 98.1 | 98.1 | 98.1 | 97.7 | 97.7 | 97.7 | 97.7 | 98.6 | 98.6 |  |  |
| 29 | *B. napus* (GU255865.1) | 77.9 | 92 | 92.3 | 92 | 92.3 | 92.3 | 92.3 | 92 | 92 | 91.8 | 91.8 | 97.9 | 97.9 | 97.9 | 97.9 | 98.1 | 98.1 | 98.3 | 98.3 | 98.3 | 98.3 | 97.8 | 97.8 | 97.8 | 97.8 | 98.8 | 98.8 | 99.8 |  |
| 30 | *B. napus* (Da-Ae-C09) | 77.9 | 92 | 92.3 | 92 | 92.3 | 92.3 | 92.3 | 92 | 92 | 91.8 | 91.8 | 97.9 | 97.9 | 97.9 | 97.9 | 98.1 | 98.1 | 98.3 | 98.3 | 98.3 | 98.3 | 97.8 | 97.8 | 97.8 | 97.8 | 98.8 | 98.8 | 99.8 | 100 |

**SUPPLEMENTARY TABLE 3 |** Details of different *TT8 gene* sequences obtained from *A. thaliana* and different *Brassica species*

| **S.N.** | **Brassica species** | **Cultivar** | **Gene ID** | **Gene Location** | **CDS length** | **Amino acids** | **References** |
| --- | --- | --- | --- | --- | --- | --- | --- |
| 1. | *A. thaliana* | Columbia | NM_117050.3 | Chromosome 4 | 1557 | 518 | Mayer et al. (1999) |
| 2. | *B. rapa* | Chiffu | GU255866.1 | A09 | 1566 | 521 | NCBI Genbank |
|  |  | Chiifu-401-42 | XM_009115326.3 | A09 | 1566 | 521 | NCBI Genbank |
|  |  | Tsuda | HQ337791.1 | A09 | 1566 | 521 | Wang et al. (2012) |
| 3. | *B. nigra* | YZ12151 | NA | B03 | 1554 | 517 | NA |
|  |  | Sangam | NA | B03 | 1554 | 517 | NA |
| 4. | *B. oleracea* | C10 | GU255867.1 | C09 | 1551 | 516 | NCBI Genbank |
|  |  | Stovepipe | GU219990.1 | C09 | 1551 | 516 | Chiu et al. (2010) |
| 5. | *B. juncea* | Tumida | KJ942581.1 | A09 | 1566 | 521 | Xie et al. (2014) |
|  |  | DRMRIJ-31 | NA | A09 | 1566 | 521 | NA |
|  |  | Varuna | NA | A09 | 1566 | 521 | Padmaja et al. (2014) |
|  |  | T84-66 | NA | A09 | 1566 | 521 | NA |
|  |  | RLC-3 | NA | A09 | 1548 | 515 | NA |
|  |  | Heera | NA | A09 | 1548 | 515 | Padmaja et al. (2014) |
|  |  | AU-213 | NA | A09 | 1548 | 515 | NA |
|  |  | Sichuan Huangzi | NA | A09 | 1548 | 515 | NA |
|  |  | DRMRIJ-31 | NA | B03 | 1554 | 517 | NA |
|  |  | T84-66 | NA | B08 | 1554 | 517 | NA |
|  |  | Varuna | NA | B03 | 1554 | 517 | Padmaja et al, (2014) |
|  |  | RLC-3 | NA | B03 | 1530 | 509 | NA |
|  |  | Heera | NA | B03 | 1530 | 509 | Padmaja et al, (2014) |
|  |  | AU-213 | NA | B08 | 1530 | 509 | NA |
|  |  | Sichuan Huangzi | NA | B08 | 1530 | 509 | NA |
| 6. | *B. napus* | J9707 | NM_001315974.2 | A09 | 1566 | 521 | Zhai et al. (2020) |
|  |  | Darmor-bzh | GU255864.1 | A09 | 1566 | 521 | NCBI Genbank |
|  |  | Da-Ae | NA | A09 | 1566 | 521 | NA |
|  |  | Darmor-bzh | GU255865.1 | C09 | 1551 | 516 | NCBI Genbank |
|  |  | Da-Ae | NA | C09 | 1551 | 516 | NA |
| 7. | *B. carinata* | sxm20200214 | NA | B01 | 1554 | 517 | NA |
|  |  | sxm20200214 | NA | C04 | 1551 | 516 | NA |
